# Design and Optimization of a Novel Three-Dimensional Force Sensor with Parallel Structure

**DOI:** 10.3390/s18082416

**Published:** 2018-07-25

**Authors:** Guanyu Huang, Dan Zhang, Sheng Guo, Haibo Qu

**Affiliations:** 1 School of Mechanical, Electronic and Control Engineering, Beijing Jiaotong University, Beijing 100044, China; 14116353@bjtu.edu.cn (G.H.); shguo@bjtu.edu.cn (S.G.); hbqu@bjtu.edu.cn (H.Q.); 2 Lassonde School of Engineering, York University, Toronto, ON M3J 1P3, Canada

**Keywords:** force sensor, parallel mechanism, performance analysis, optimization

## Abstract

To measure large external forces exerted on a loading platform, a novel three-dimensional force sensor is developed in this paper. The proposed sensor was designed with a parallel mechanism with three degrees of freedom. Kinematic analysis of this sensor was performed. Due to its structural characteristics, the working principle of the sensor was analyzed using a Jacobian matrix. The sensitivity diversity index and measuring capability were both calculated. The analysis showed that the proposed sensor is more suitable for measuring large forces than existing strain sensors. In addition, compared with existing strain sensors, this sensor is more suitable for measuring forces along the *x* and *y* axes. By changing the stiffness coefficients of the springs, the proposed sensor has reconfigurability. This sensor can change its measuring capability to meet different requirements. Next, the mode shapes and natural frequencies of the proposed sensor were performed. Finally, based on these performance indices, the design variables were optimized using a Multi-Objective Genetic Algorithm.

## 1. Introduction

Force measurement is a critical requirement in many fields [1], including intelligent control [2], medical operations [3,4], and rehabilitation appliances [5,6]. To measure force, many force sensors have been introduced. Compared with one-dimensional (1D) force sensors, many researchers have focused on multi-dimensional force and torque sensors. For a large-load robotic manipulator, Li et al. [7,8] proposed a novel piezoelectric six-dimensional (6D) large force and moment sensor. The characteristic dynamic vibration modes of the proposed sensor were extracted by analyzing special experimental data. Valdastri et al. [9] proposed and characterized a novel hybrid silicon three-axial force sensor that was developed for biomechanical measurements. To measure vibrissal contacts, Quist et al. [10] developed a simple yet effective two-dimensional (2D) force sensor with ±0.02 mN resolution.

Due to their mobility, some forms of parallel sensing mechanisms are suitable as multi-dimensional force sensors. These sensors also have the advantages of stability, high loading capability, zero error accumulation, and high accuracy [11]. By combining a parallel mechanism with integrated flexible joints, Zhao et al. [12] proposed a highly accurate sensor with a large measurement range. Then, based on a flexible joint 6-UPUR parallel six-axis force sensor, the authors performed assembly and deformation error modeling and analyzed the large measurement range and high accuracy of the resulting sensors. Song et al. [13] developed a novel four degrees of freedom (DOF) wrist force and torque sensor to measure the multi-dimensional interactive force between the human hand and an interaction device. The sensor was made of two elastic beams that can be viewed as the parallel structure. Based on a six-DOF compliant parallel mechanism, Liang et al. [14] developed a micro-scale sensor with high precision that could provide real-time force information for feedback control. This sensor featured piezo-driven actuators and an integrated force sensor capable of delivering six-DOF motions. Based on the parallel mechanism, Gao and Zhang [15] proposed a novel acceleration sensor. This sensor was a novel 3-RRPRR fully decoupling parallel mechanism. Then, by using a self-developed calibration platform, its sensitivity and linearity were verified. To measure the ground reaction force of a human or humanoid robot, Nishiwaki et al. [16] developed a six-axis force sensor. By using a parallel support mechanism, this sensor allows large torques or forces. Based on a 3-RRR parallel micro-motion stage, a new force sensor was proposed by Hu et al. [17]. This sensor can be used in precision engineering, such as micro-force and torque measurement. Based on the modified Stewart platform, Yao et al. [18] proposed the structural model of a generalized redundant parallel six-axis force sensor. For some typical redundant six-axis force sensors, mathematical models were established for the corresponding structures.

Sensitivity and measuring capability are the significant indices for these sensors. For parallel sensors, the relationship between design variables and performance is inseparable. To create a sensor with better performance, optimization algorithms are widely used [19]. For a novel hyperstatic six-component force and torque sensor, Hou et al. [20] applied genetic algorithms (GAs) to optimize the performance of the proposed sensor. Sun et al. [21,22] proposed a novel six-axis force and torque sensor for a space robot; response surface methodology was used to determine the optimum dimensional parameters. To improve the performance of the parallel six-axis force sensor with a Stewart platform, Zhao et al. [23] developed the nonlinear single objective and multi-objective algorithm. Sun et al. [24] developed a particle swarm algorithm to optimize a six-axis force and torque sensor. Also, the least square support vector machine (LSSVM) was used to achieve temperature compensation for the optimized sensor. To minimize the cross error, Kang et al. [25] proposed a new term called “principal coupling”. By using an algorithm, the performance of the mechanically decoupled six-axis force and torque sensor was improved.

Furthermore, some fields have special needs in terms of sensitivity and measuring capability. For example, measuring the three-dimensional (3D) ground reaction force (GRF) in the human gait [26] requires more sensitivity along the *x* and *y* axes than along the *z*-axis. Sometimes, reconfigurability is also a special requirement for the sensor [27,28]. In this study, we develop a novel force sensor with a parallel structure. Based on its structure, the loading force can only move along the three axes and the proposed sensor can measure large forces. By measuring the linear encoders added to the prismatic joints, the force exerted on the loading platform can be calculated. Based on its structure, this sensor is more sensitivity along the *x* and *y* axes than along the *z*-axis. Notably, the proposed sensor can change its measuring capability by changing spring with different stiffness coefficients. Due to its application, the design objectives of the proposed 3D force sensor with a parallel structure are given as following, the goal range along *x, y*-axis should be [−800 N, 800 N] and the goal range along *z*-axis should be [0 N, 400 N]. Considering the requirement of sensitivity, the sensitivity along the *x* and *y* axes should be higher than it along *z*-axis.

The rest of this paper is organized as follows: the model is described in Section 2, and the kinematic analysis is provided in Section 3. The performance analysis, including sensitivity diversity and measuring capability, are both discussed in Section 4. The Multi-Objective Genetic Algorithm is performed in Section 5. Finally, we conclude our work in Section 6.

## 2. Model Description and Mobility Analysis

**Figure 1:**
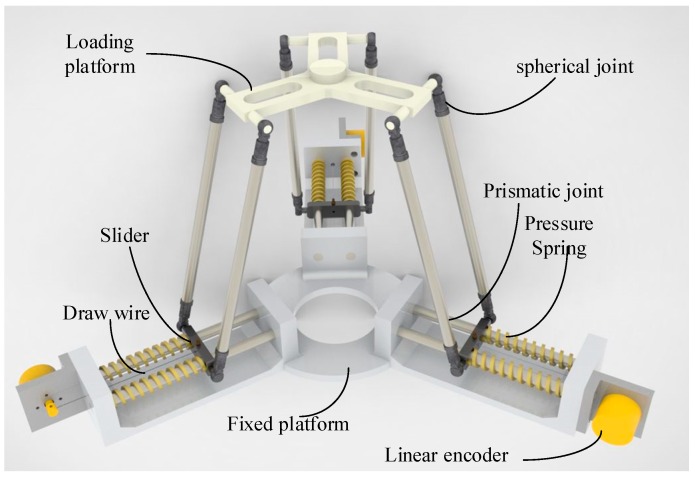
The computer-aided design (CAD) model of proposed force sensor.

The proposed 3D force sensor is shown in Figure 1. The proposed sensor is structured with a parallel mechanism. The sensor has three identical limbs and each limb has a prismatic joint—a parallelogram mechanism. The upper link of the parallelogram mechanism belongs to the loading platform, and the lower link is the slider connected to the fixed platform by a prismatic joint. All the joints in parallelogram mechanism are spherical joint. To optimize the structure, the joint bearings are chosen as the spherical joints. To balance the slider and attain stability, the prismatic joint includes two slide bars. Two pressure springs with a high and constant spring stiffness coefficient are added around the two slide bars. The end of the swing link, a linear encoder, is attached to measure the displacement of the prismatic joint by using a draw wire connected to the slider and encoder. When the external force, including the *x*, *y*, and *z* axes, acts on the loading platform, the prismatic joints are actuated. Then, the displacements of three prismatic joints can be measured by three linear encoders. By measuring the displacements of three prismatic joints, the external force can be calculated by a transformation equation.

**Figure 2:**
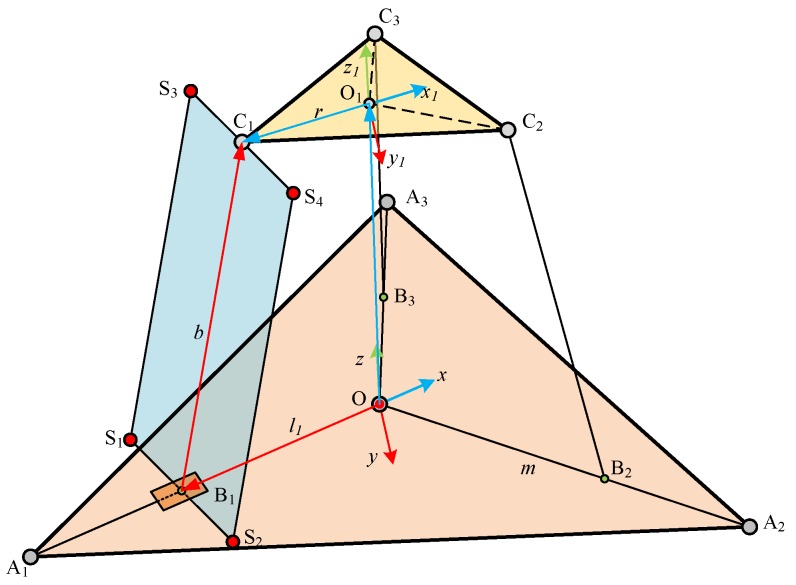
The scheme of the proposed sensor.

The scheme of the proposed sensor is shown in Figure 2. The mobility of the proposed sensor can be analyzed using the Screw Theory. The coordinates of the points $A_{1}$, $B_{1}$, and $C_{1}$ can be denoted as ${\left[\begin{array}{ccc} x_{a}^{1} & y_{a}^{1} & z_{a}^{1} \end{array}\right]}^{T}$, ${\left[\begin{array}{ccc} x_{b}^{1} & y_{b}^{1} & z_{b}^{1} \end{array}\right]}^{T}$, and ${\left[\begin{array}{ccc} x_{c}^{1} & y_{c}^{1} & z_{c}^{1} \end{array}\right]}^{T}$, respectively. Thus, the twist system of limb $A_{1}B_{1}C_{1}$ can be written as:

(1) $$\begin{array}{l} {\$}_{1}={\left[\begin{array}{cccccc} 0 & 0 & 0; & l & m & 0 \end{array}\right]}^{T}\;\\ {\$}_{2}={\left[\begin{array}{cccccc} -m & l & 0; & lz_{a}^{1} & mz_{a}^{1} & -lx_{a}^{1}-my_{a}^{1} \end{array}\right]}^{T}\;\\ {\$}_{3}={\left[\begin{array}{cccccc} a & b & c; & -cy_{b}^{1}+bz_{b}^{1} & cx_{b}^{1}-az_{b}^{1} & -bx_{b}^{1}+ay_{b}^{1} \end{array}\right]}^{T}\;\\ {\$}_{4}={\left[\begin{array}{cccccc} a & b & c; & -cy_{b}^{1}+bz_{b}^{1} & cx_{b}^{1}-az_{b}^{1} & -bx_{b}^{1}+ay_{b}^{1} \end{array}\right]}^{T}\;\\ {\$}_{5}={\left[\begin{array}{cccccc} a & b & c; & -cy_{c}^{1}+bz_{c}^{1} & cx_{c}^{1}-az_{c}^{1} & -bx_{c}^{1}+ay_{c}^{1} \end{array}\right]}^{T}\\ {\$}_{6}={\left[\begin{array}{cccccc} a & b & c; & -cy_{c}^{1}+bz_{c}^{1} & cx_{c}^{1}-az_{c}^{1} & -bx_{c}^{1}+ay_{c}^{1} \end{array}\right]}^{T}\\ {\$}_{7}={\left[\begin{array}{cccccc} -m & l & 0; & lz_{c}^{1} & mz_{c}^{1} & -lx_{c}^{1}-my_{c}^{1} \end{array}\right]}^{T}\; \end{array}$$

where l and m express the direction of prismatic joint axis, and a, b, and c express the direction of the spherical joint axis along the *x*, *y*, and *z* axes, respectively.

Thus, the wrench system of this limb can be obtained as:

(2) $${\$}^{r}={\left[\begin{array}{llllll} 0 & 0 & 0; & -\frac{cl}{al+bm} & -\frac{cm}{al+bm} & l \end{array}\right]}^{T}$$

The wrench systems of the other two limbs can be calculated using the same method:

(3) $$\begin{array}{l} {\$}_{1}^{r}={\left[\begin{array}{llllll} 0 & 0 & 0; & -\frac{c_{1}l_{1}}{a_{1}l_{1}+b_{1}m_{1}} & -\frac{c_{1}m_{1}}{a_{1}l_{1}+b_{1}m_{1}} & l_{1} \end{array}\right]}^{T}\\ {\$}_{2}^{r}={\left[\begin{array}{llllll} 0 & 0 & 0; & -\frac{c_{2}l_{2}}{a_{2}l_{2}+b_{2}m_{2}} & -\frac{c_{2}m_{2}}{a_{2}l_{2}+b_{2}m_{2}} & l_{2} \end{array}\right]}^{T} \end{array}\;$$

Based on Equations (2) and (3), the overall twist system of the sensor can be calculated as:

(4) $$\$=\{\begin{array}{l} {\left[\begin{array}{llllll} 0 & 0 & 0; & 0 & 0 & 1 \end{array}\right]}^{T}\\ {\left[\begin{array}{llllll} 0 & 0 & 0; & 0 & 1 & 0 \end{array}\right]}^{T}\\ {\left[\begin{array}{llllll} 0 & 0 & 0; & 1 & 0 & 0 \end{array}\right]}^{T} \end{array}$$

With Equation (4), the three-DOF plane movement mobility of this sensor can be analyzed.

## 3. Kinematics Analysis

As shown in Figure 2, the sensor has three identical limbs and the angles between arbitrary neighborhood limbs are all 120°. The fixed platform is located on the ground and the fixed coordinate system denoted as Oxyz assumes that the *x*-axis is along the vector $\vec{A_{1}O}$. The original point of the moving coordinate system is fixed at the center of the loading platform, denoted as $O_{1}x_{1}y_{1}z_{1}$, and the *x*-axis is along the vector $\vec{C_{1}O_{1}}$. To analyze the kinematics, some structural parameters are provided: $\left\|\vec{OB_{i}}\right\|=l_{i}$, $\left\|\vec{OA_{i}}\right\|=m$, $\left\|\vec{B_{i}C_{i}}\right\|=b$, and $\left\|\vec{C_{i}O_{i}}\right\|=r$. When the sensor is working, assume the external force are known, and then the position of the loading platform will change. Namely, the coordinate of $O_{1}$ is known, denoted as ${\left[\begin{array}{ccc} x & y & z \end{array}\right]}^{T}$. The lengths of the three prismatic joints are regarded as the unknown variables. 

The closed-loop vector equation of the sensor can be written as:

(5) $$\vec{OB_{i}}+\vec{B_{i}C_{i}}=\vec{OO_{i}}+\vec{O_{i}C_{i}}$$

Given Equation (4), the sensor only has three translational degrees of freedom. Thus, the transformation matrix between the loading platform and fixed platform can be written as:

(6) $$R=\left[\begin{array}{ccc} 1 & 0 & 0\\ 0 & 1 & 0\\ 0 & 0 & 1 \end{array}\right]\;$$

The coordinate of point $C_{i}\left(i=1,2,3\right)$ in the fixed coordinate system can be written as:

(7) $$C_{i}=\left[\begin{array}{c} x+r\cos\theta_{i}\\ y+r\sin\theta_{i}\\ z \end{array}\right]\;$$

Then, the coordinate of point $B_{i}\left(i=1,2,3\right)$ in the fixed coordinate system can be written as:

(8) $$B_{i}=\left[\begin{array}{c} l_{i}\cos\theta_{i}\\ l_{i}\sin\theta_{i}\\ 0 \end{array}\right]$$

where $\theta_{i}\left(i=1,2,3\right)$ is the angle between two neighboring limbs.

Due to the geometrical constraint of the sensor, the length of link $\left\|\vec{B_{i}C_{i}}\right\|$ is fixed. Thus, substituting Equations (7) and (8) into Equation (5), the lengths of prismatic joints can be solved as:

(9) $$l_{i}=r+\csc\theta_{i}\left(y+\csc\theta \sqrt{{\left(\sin\theta_{i}\right)}^{2}\left(b^{2}-x^{2}-z^{2}-\left(r-1\right)\left(2x+\left(r-1\right)\cos\theta_{i}\right)\cos\theta_{i}\right)}\right)\left(i=1,2,3\right)\;$$

**Figure 3:**
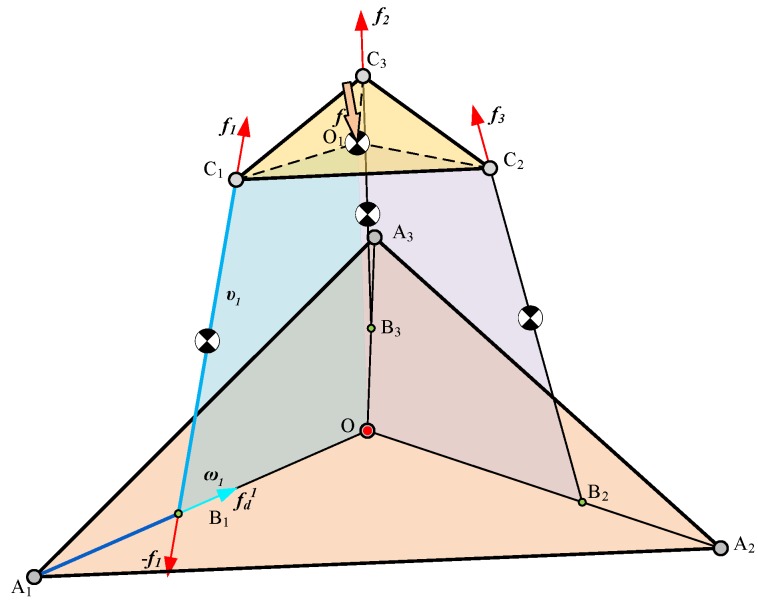
The scheme of the loading force.

For this sensor, the relationship between the loading force and the lengths of the prismatic joints is very important. As shown in Figure 3, the loading force is denoted as f and the vector of limb $\vec{B_{i}C_{i}}$ is denoted as $\upsilon_{i}\left(i=1,2,3\right)$. The vector of limb $\vec{A_{i}O}$ is denoted as $\omega_{i}\left(i=1,2,3\right)$. Due to the principles of static equilibrium, the external force acting on the loading platform can be written as:

(10) $$\sum_{i=1}^{3}f_{i}\upsilon_{i}+\left(f+m_{1}g\right)=0\left(i=1,2,3\right)$$

where $m_{1}$ is the mass of the loading platform and g is the gravity acceleration.

The limb $C_{i}B_{i}$ can be regarded as a binary link; thus, the force acting on the spring and driving linear encoder can be calculated as:

(11) $$\left(f_{i}+m_{2}g\right)\upsilon_{i}=f_{d}^{1}\omega_{1}\left(i=1,2,3\right)$$

where $m_{2}$ is the mass of the parallelogram mechanism.

Assuming that $\kappa_{i}$ is the stiffness coefficient of the spring and $\Delta l_{i}\left(i=1,2,3\right)$ is the displacement measured by the linear encoder, yielding:

(12) $$f_{d}^{1}\omega_{1}=\kappa_{i}\left(L+l_{i}-m\right)\left(i=1,2,3\right)\;$$

where L is the length of the pressure spring and $\kappa_{i}$ is determined by:

(13) $$\kappa_{i}=\frac{Gd^{4}}{8N{\left(D_{o}-d\right)}^{3}}$$

where G is the modulus of rigidity, d is the wire diameter, N is the active coil number, and $D_{o}$ is the outside diameter.

Based on Equations (10)–(12), the relationship between the external force acting on the loading platform and the spring and linear encoder can be calculated.

In general, the external forces include three directions: the three component forces along the *x*-, *y*-, and *z*-axis, which can be denoted as $f={\left[\begin{array}{ccc} f_{x} & f_{y} & f_{z} \end{array}\right]}^{T}$. Thus, the displacements of the three linear encoders can be used to estimate the external force by the following equation:

(14) $$\Delta l_{i}=J\left(f_{x},f_{y},f_{z}\right)\left(i=1,2,3\right)$$

## 4. Performance Analysis

### 4.1. Sensitivity Diversity Index

Sensitivity is an important aspect of a sensor, which can be evaluated as the rate of variation between the input and output. Our sensor was designed using a parallel mechanism. Thus, for this sensor, the sensitivity of the sensor was the rate of variation between the loading force and the output displacements. To simply this problem, the working model of the sensor can be viewed as a statics analysis of the parallel mechanism. The proposed sensor only has three translation degrees of freedom, so the virtual work principle can be applied, but the Jacobian matrix should be calculated first. In this paper, the Jacobian matrix was obtained using Screw Theory.

In a fixed coordinate system, the instantaneous twist of the moving platform can be expressed by ${＄}_{p}={\left[w\;v\right]}^{T}$, where w is the angular velocity and v is the linear velocity. Each limb can be seen as a PUU limb. Thus, the twist system can be written as:

(15) $${＄}_{P}^{1}={\dot{l}}_{11}{＄}_{11}+{\dot{\theta }}_{12}{＄}_{12}+{\dot{\theta }}_{13}{＄}_{13}+{\dot{\theta }}_{14}{＄}_{14}++{\dot{\theta }}_{15}{＄}_{15}$$

where ${＄}_{1i}$ is the unit twist screw of the *i*th joint, and ${\dot{l}}_{1i}$ or ${\dot{\theta }}_{11}$ is the linear or angular velocity of the *i*th joint, respectively. A constraint screw ${＄}_{1r}$ that is reciprocal to all the joint screws can be given as:

(16) $${＄}_{P}^{1}\circ {＄}_{1r}=0$$

Therefore, for the overall sensor, the constraint Jacobian matrix can be written as:

(17) $$J_{c}\circ {＄}_{P}^{i}\;=\;0$$

where:

(18) $$J_{c}=\left[\begin{array}{cc} 0_{3\times 1} & \tau_{1}^{T}\\ 0_{3\times 1} & \tau_{2}^{T}\\ 0_{3\times 1} & \tau_{3}^{T} \end{array}\right]$$

where $J_{c}$ is the constraint Jacobian matrix of the sensor.

However, a screw ${＄}_{1r}^{1}$ that is reciprocal to all the joint screws, except for the prismatic joint screw, can be found, which is the force exerted by the actuated joint. This screw can be given as:

(19) $${＄}_{1r}^{1}\circ {＄}_{P}^{1}={\dot{l}}_{1}\;$$

Thus, for the overall sensor, this Jacobian matrix can be written as:

(20) $$J_{a}\circ {＄}_{p}^{i}={\dot{q}}_{a}$$

where:

(21) $$J_{a}=\left[\begin{array}{cc} n_{1}^{T} & {\left(m_{1}\times n_{1}\right)}^{T}\\ n_{2}^{T} & {\left(m_{2}\times n_{2}\right)}^{T}\\ n_{3}^{T} & {\left(m_{3}\times n_{3}\right)}^{T} \end{array}\right]$$

(22) $${\dot{q}}_{a}={\left[\begin{array}{ccc} {\dot{l}}_{1} & {\dot{l}}_{2} & {\dot{l}}_{3} \end{array}\right]}^{T}$$

where $J_{a}$ is the prismatic joints’ Jacobian matrix.

Casting Equations (18) and (21) into matrix-vector form, the result can be simplified as:

(23) $$\left[\begin{array}{c} \begin{array}{cc} 0_{3\times 1} & \tau_{1}^{T}\\ 0_{3\times 1} & \tau_{2}^{T}\\ 0_{3\times 1} & \tau_{3}^{T} \end{array}\\ \begin{array}{cc} n_{1}^{T} & {\left(m_{1}\times n_{1}\right)}^{T}\\ n_{2}^{T} & {\left(m_{2}\times n_{2}\right)}^{T}\\ n_{3}^{T} & {\left(m_{3}\times n_{3}\right)}^{T} \end{array} \end{array}\right]\left[\begin{array}{c} {＄}_{p}^{1}\\ {＄}_{p}^{2}\\ {＄}_{p}^{3}\\ {＄}_{p}^{1}\\ {＄}_{p}^{2}\\ {＄}_{p}^{3} \end{array}\right]=\;\dot{q}$$

where $\dot{q}\;=\;{\left[\begin{array}{cccccc} 0 & 0 & 0 & {\dot{l}}_{1} & {\dot{l}}_{2} & {\dot{l}}_{3} \end{array}\right]}^{T}$.

The overall Jacobian matrix can then be given as:

(24) $$J={\left[\begin{array}{c} \begin{array}{cc} 0_{3\times 1} & \tau_{1}^{T}\\ 0_{3\times 1} & \tau_{2}^{T}\\ 0_{3\times 1} & \tau_{3}^{T} \end{array}\\ \begin{array}{cc} n_{1}^{T} & {\left(m_{1}\times n_{1}\right)}^{T}\\ n_{2}^{T} & {\left(m_{2}\times n_{2}\right)}^{T}\\ n_{3}^{T} & {\left(m_{3}\times n_{3}\right)}^{T} \end{array} \end{array}\right]}^{-1}$$

Let the loading force be denoted by $f\;=\;{\left[\begin{array}{ccc} f_{x} & f_{y} & f_{z} \end{array}\right]}^{T}$ and let the vector of joint forces be denoted by $f_{j}={\left[\begin{array}{ccc} f_{1} & f_{2} & f_{3} \end{array}\right]}^{T}$. This sensor is designed for the large force, due to its application. Thus, assume the frictional forces at the joints are negligible and the virtual work produced by the constraint forces at the joints is zero. Hence, the virtual work completed by all the linear springs is given by:

(25) $$\delta w=f_{j}{}^{T}\delta q-\left(f^{T}+G\right)\delta x$$

where G is the mass matrix of the loading platform and the three parallelogram mechanisms, and δq and δx are the virtual displacements of the measuring joints and loading platform, respectively.

Based on Equation (23), the virtual displacements δq and δx are not independent; they are related by the Jacobian matrix as follows:

(26) δx=Jδq

Substituting Equation (26) into Equation (25) yields:

(27) $$f_{j}{}^{T}\delta q-\left(f^{T}+G\right)J\delta q=0$$

Thus, calculating Equation (27) yields:

(28) $$f_{j}=J^{T}\left(f+G^{T}\right)$$

The measurement sensitivities of the sensor with exerting forces along *x*, *y* and *z*-axis are defined as the eigenvalues of the Jacobian matrix, which are:

(29) $$J^{T}=\left[\begin{array}{ccc} J_{x} & 0 & 0\\ 0 & J_{y} & 0\\ 0 & 0 & J_{z} \end{array}\right]\;$$

The variation in the sensor sensitivity for forces exerted in different axes is the sensitivity diversity index, which can be calculated as:

(30) $$\upsilon_{d}=\sqrt{\frac{\lambda_{\max}}{\lambda_{\min}}}\;$$

where $\lambda_{\max}$ and $\lambda_{\min}$ are the largest and smallest eigenvalues of the Jacobian matrix, respectively.

And the sensitivities of the sensor for loading force in the *x*, *y*, *z*-axis can be defined as the eigenvalues of each column in the Jacobian matrix.

### 4.2. Measuring Capability

**Figure 4:**
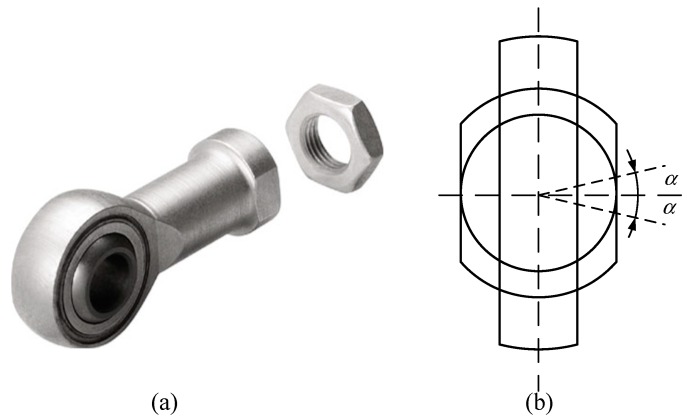
The spherical joint: (**a**) CAD model, (**b**) sketch of the joint.

The measuring capability of the proposed sensor is a significant performance index. The sensor prototype involves a parallel mechanism, so the measuring capability was determined by the workspace of the loading platform and the stiffness coefficients of the springs. The workspace of the sensor was mainly constrained by the spherical joint and the range of the prismatic joint. As shown in Figure 4, the range of the spherical joint is [−13°,13°]. Taking $∠s_{s}s_{4}s_{2}$ as an example, the angle of the joint can be calculated as:

(31) $$\alpha =\arccos\frac{\vec{s_{3}s_{4}}\cdot \vec{s_{2}s_{4}}}{\left\|\vec{s_{3}s_{4}}\right\|\cdot \left\|\vec{s_{s}s_{4}}\right\|}\;$$

Similar to Equation (31), the other angles can be calculated using the same method. The range in the prismatic joint is determined by the pressure spring and the structural parameters. As such, the range can be written as:

(32) $$m-L\leq l_{i}\leq m-Nd\left(i=1,2,3\right)\;$$

In this paper, the material of the pressure spring is spring steel (SUP) and the structural parameters of the sensor are listed in Table 1.

Given the parameters in Table 1, the stiffness coefficient of the spring can be calculated as *k_i_* = 16.5343 N/mm. The constraint of the prismatic joint can be obtained as:

(33) $$190\;\mathrm{mm}\leq l_{i}\leq 240\;\mathrm{mm}\;$$

**Figure 5:**
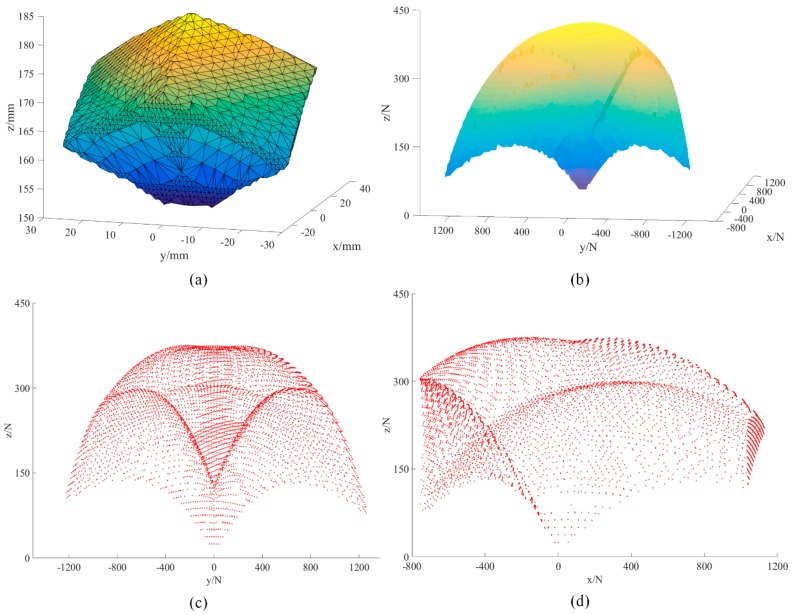
(**a**) Workspace of the parallel mechanism, (**b**) measuring capability of the sensor, (**c**) measuring distribution when fx = 0 N, and (**d**) measuring distribution when fy = 0 N.

Based on Equations (29) and (33), the measuring capability of the sensor can be calculated. The result is shown in Figure 5. As shown in the figure, both the workspace and the measuring capability are triangular symmetrical, just like the configuration of the proposed sensor. The measuring range along the *x*, *y*, and *z* axes are [−800 N, 1200 N], [−1200 N, 1200 N], and [0 N, 410 N], respectively. The proposed sensor has a larger measuring range along the *x* and *y* axes than along the *z*-axis. Furthermore, this figure also shows that the proposed sensor is more suitable for measuring shear force than the existing strain force.

**Figure 6:**
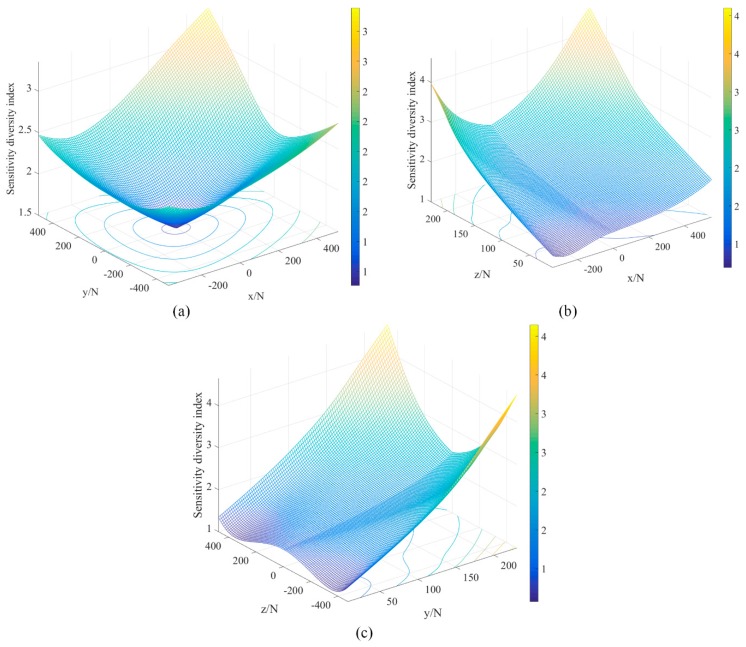
The sensitivity diversity index in different planes: (**a**) plane *xoy*, (**b**) plane *xoz*, and (**c**) plane *yoz.*

The sensitivity diversity indices of planes xoy, xoz, and yoz are shown in Figure 6. The indices in plane xoy are better than the indices in planes xoz or plane yoz, which means that the sensor has sensing ability in the horizontal direction than in the vertical direction. Based on the performance analysis, the proposed sensor has a greater performance along the *x* and *y* axes than along the *z*-axis. 

**Figure 7:**
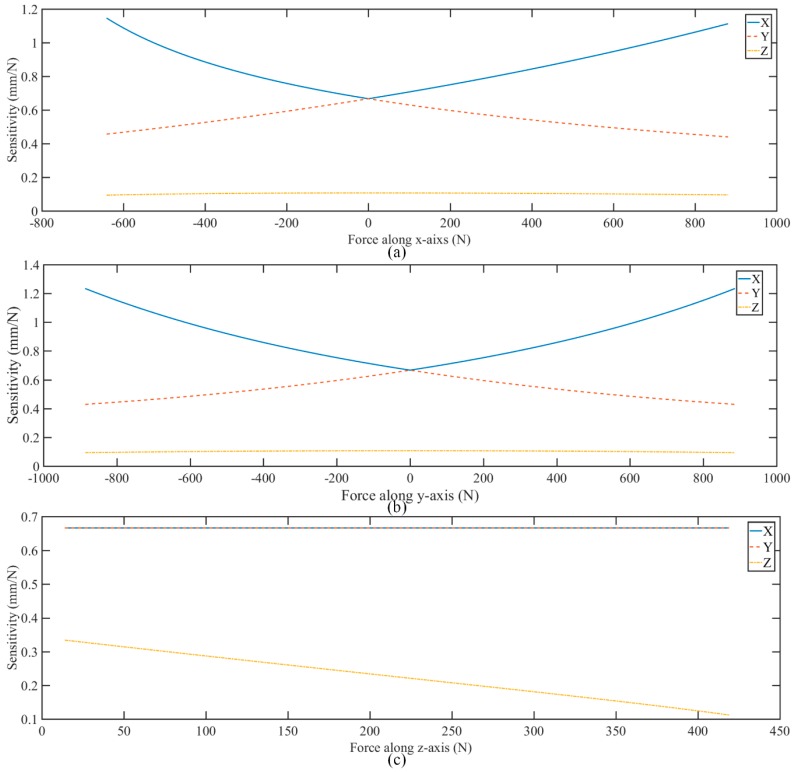
The sensitivity along different axes: (**a**) *x*-axis, (**b**) *y*-axis, (**c**) *z*-axis.

Similar to sensitivity diversity index, the sensitivity along *x*, *y* and *z*-axis are shown in Figure 7. The sensitivities along *x*, *y*-axis are both better than sensitivity along z-axis. Namely, the sensor has better sensitivity in the horizontal direction than in the vertical direction. In this paper, the traditional force sensor can be defined as following: (a) the structure is monolithic; (b) the principle is measuring the deformation of strain gages; (c) the type is strain sensor. The comparisons of sensitivity and measuring range along *x*, *y z*-axis between existing strain sensors and proposed sensor are list in Table 2. To make the contrast even more remarkable, the sensitivity and measuring range along *z*-axis are both as 1. The sensitivity and measuring range along *x*, *y*-axis is set as the ratios to *z*-axis. Compared with other strain sensors, the proposed sensor is more suitable for measuring shear force.

**Figure 8:**
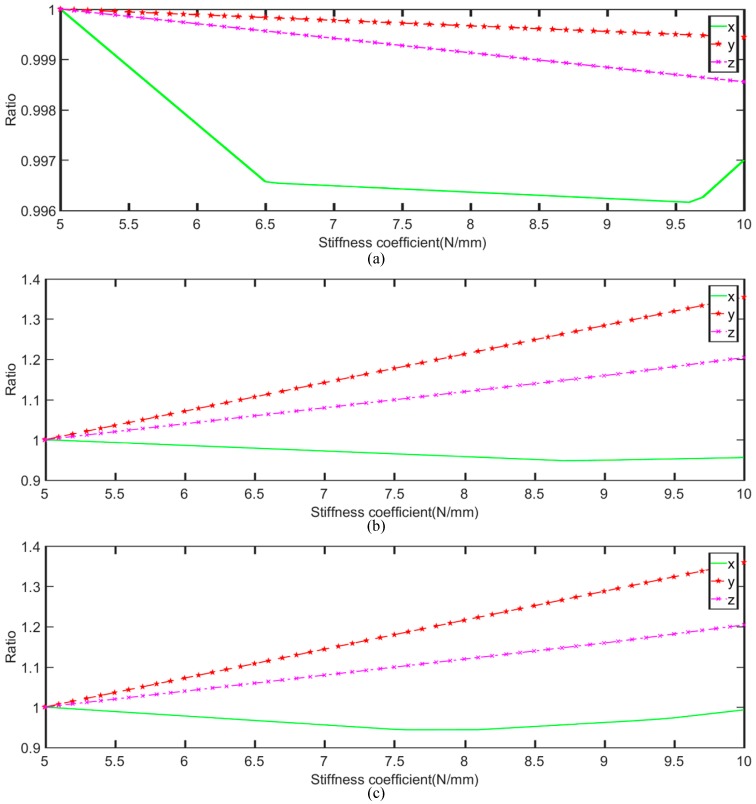
The measuring range trend of the proposed sensor with different spring stiffness coefficients: (**a**) the first prismatic joint, (**b**) the second prismatic joint, and (**c**) the third joint.

The significant feature of the proposed sensor is that the performance of the sensor can be changed by using springs with different stiffness coefficients. When the stiffness coefficients are changing, the effective measuring range of the *x*, *y*, and *z* axes will be different. Therefore, in this section, the stiffness coefficient of one limb is changed and the coefficients of the other two limbs are constant, and the result of the effective measuring range is shown in Figure 8. As shown in the figure, for the first joint, when the coefficient increases, the ranges of the *x*, *y*, and *z* axes all decrease. However, the variation is small. For the second and third joints, the coefficients are similar. When the coefficient increases, the ranges of the *y*, and *z* axes increase, but the range of the x-axis decreases. Therefore, based on this result, the effective measuring capability can be determined by choosing different coefficients for different joints. Specifically, the proposed sensor can change its measuring capability by adding a different spring to the prismatic joints. Thus, the proposed sensor is a reconfigurable sensor.

### 4.3. Dynamic Analysis

When the proposed sensor is working, the sensor actually behaves dynamically. So a dynamic model has been established via ANSYS to confirm that the proposed sensor has a good dynamic performance such as resonance frequency and mode shapes, which are regarded as the important indices in the design of a structure for dynamic loading conditions [29].

**Figure 9:**
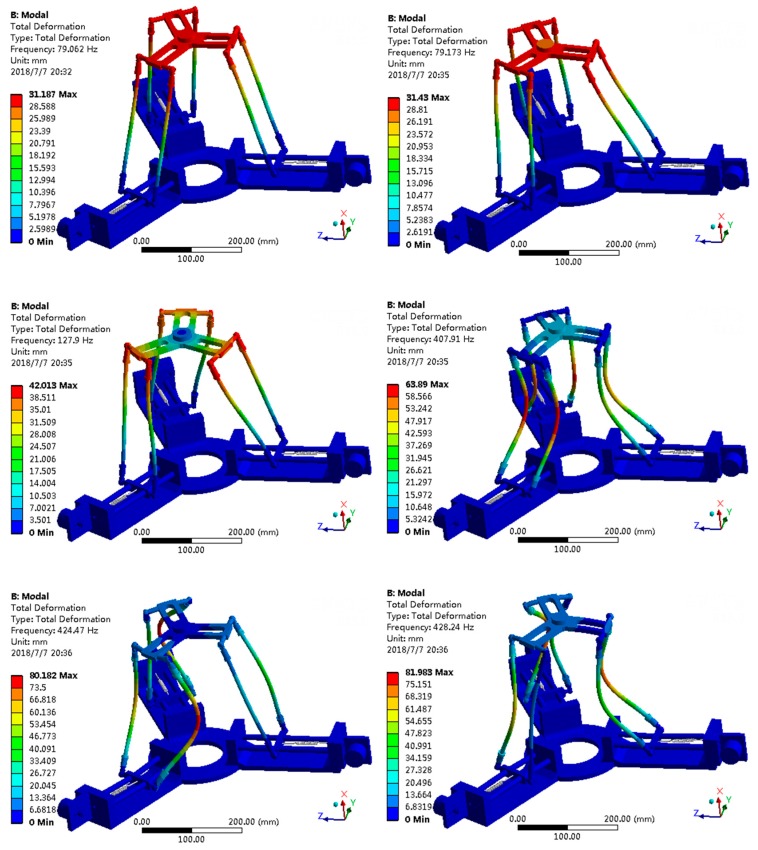
Mode shapes of the proposed sensor.

The fixed platform of the proposed sensor is fixed on the ground, when the loading platform is connected with manipulators, and the first six natural frequencies of the proposed sensor has been specified. Finally, the first six natural frequencies are listed in Table 3, and the corresponding mode shapes pictures are shown in Figure 9. Table 3 and Figure 9 are helpful in understanding how the sensor vibrates. The first and second, the fifth are sixth mode shapes are both similar, which means that they are approximately along *x*-axis and *y*-axis, respectively. And the third and fourth mode shapes are approximately moving along *z*-axis.

## 5. Multi-Objective Optimization

### 5.1. Fitness Function

In this section, based on the Multi-Objective Genetic Algorithm, the design variable is optimized. The purpose of the optimization was to obtain a modified sensor with better performance [32]. The first step was to determine the fitness functions. Given the above analysis, the sensitivity diversity index and measuring capability were both important indices for the proposed sensor. Thus, they were regarded as the fitness functions.

Based on Equation (30), the sensitivity diversity index is a local performance index used to evaluate the global performance of the proposed sensor. Therefore, a global sensitivity diversity index (GSDI) is defined as:

(34) $$\mathrm{GSDI}=\frac{\int \upsilon_{d}dW}{\int dW}$$

where:

(35) ∫dW=∭dxdydz

In addition to the global sensitivity diversity index (GSDI), measuring capability was also an important factor affecting the performance of the proposed sensor. As such, the fitness function was determined by the global sensitivity diversity index and measuring capability. To obtain the best performance, the measuring capability should be higher and the global sensitivity diversity index should be as low as possible.

### 5.2. Optimization Results

**Figure 10:**
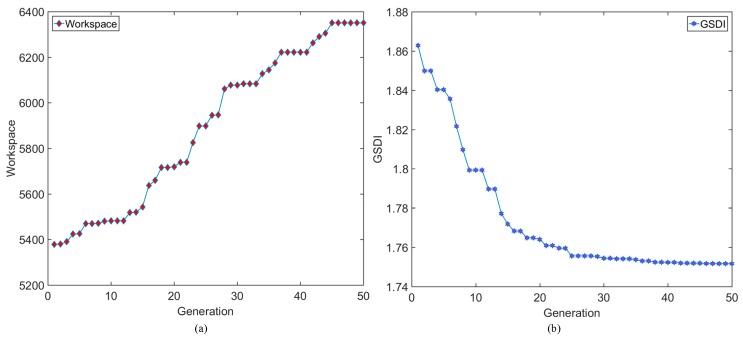

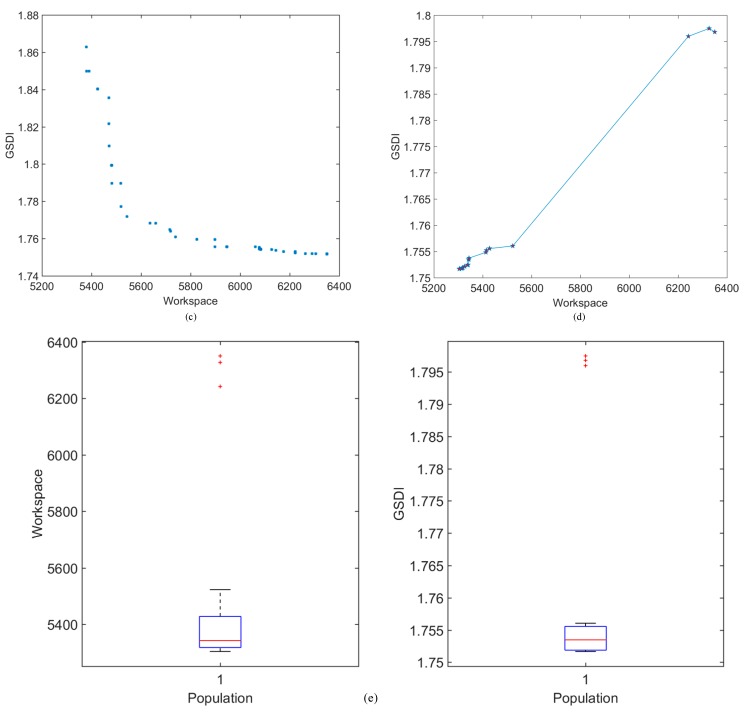
The result of the Multi-Objective Genetic Algorithm (MOGA): (**a**) the workspace results, (**b**) the GSDI results, (**c**) evolution of the population, (**d**) Pareto Frontier of the best individual, and (**e**) box-plot of the workspace and GSDI.

Based on the Multi-Objective Genetic Algorithm (MOGA), the optimized parameters were set as *m*, *b*, and *r*. For practical application, the variable constraints should be determined, which are listed in Table 4. During the optimization process, the population was set to 20, the crossover probability was set to 0.9, and the mutation probability was set as 0.05. Considering the efficiency and computing capability, the number of generations was set to 50. The result of the MOGA is shown in Figure 10. Figure 10a,b are the results of the workspace and GSDI. At the end of the optimization, the values of the workspace and GSI were both stable. Additionally, the performance indices were both better than the initial parameters. Figure 10c shows the trend in the average value of the workspace and GSDI in the optimal process. The Pareto Frontier is shown in Figure 10d. Figure 10e shows the values of the workspace and GSI of the last population. Based on Figure 10, the parameters of the proposed sensor were optimized by regarding the global sensitivity diversity index and measuring capability as the fitness functions. Due to the optimized solutions, the suitable parameters can be chosen for special task.

Example objective function values and corresponding design variables are listed in Table 5. Due to the requirements of task, the design variables for the proposed sensor were chosen.

## 6. Conclusions

To measure forces along different axes, a novel force sensor was proposed that uses a parallel mechanism. Based on Screw Theory, its mobility is analyzed. Due to the closed-loop vector equation, a kinematic model of the proposed sensor was established. Due to its parallel structure, the working principle of the proposed sensor is the static equilibrium of the parallel mechanism. Thus, by using a Jacobian matrix, the sensitivity diversity index and measuring capability were calculated. In addition, the results of the sensitivity diversity index and measuring capability showed that the sensor has a greater performance along the *x* and *y* axes than the *z*-axis. Notably, the proposed sensor is better than existing strain sensors for measuring horizontal force. Furthermore, based on its parallel structure, the measuring capability showed that the proposed sensor is more suitable for a large force or a large load field. Next, the relationship between the performance of the proposed sensor and the stiffness coefficient of springs was discussed, and the mathematic model was established. The results showed that the proposed sensor can change its measuring capability and sensitivity diversity by adjusting the stiffness coefficient of the springs. Notably, the proposed sensor has reconfigurability. Next, the first six nature frequencies and mode shapes of the proposed sensor were performed by using ANSYS, and the results can be regarded as the dynamic criteria to apply this sensor.

Based on the Multi-Objective Genetic Algorithm, the design variables of the proposed sensor were optimized, using measuring capability and sensitivity diversity as the fitness functions. Compared with the initial design variables, the workspace of the optimized sensor increased by 18.05% and the GSDI of the optimized sensor increased by 6.35%. Due to the provided solution, the suitable design variables can be chosen to meet different requirements.

## Figures and Tables

**Table 1:** The parameters of the proposed sensor.

Parameter	Value	Parameter	Value (mm)
*G*	8000 kg/mm^2^	*r*	92
*d*	2.5	*b*	208
*D_o_*	14	*L*	90
*N*	20	*m*	280

**Table 2:** Comparison with existing strain sensors.

	Range along *x*-Axis	Range along *y*-Axis	Sensitivity along *x*-Axis	Sensitivity along *y*-Axis
Liang et al., (2009) [29]	1.2	1.2	0.73	0.57
Liang et al., (2010) [30]	1.4	1.4	1.07	1.15
Song et al., (2007) [13]	1	1	1.4	1.4
Wu et al., (2011) [31]	0.53	0.53	0.69	0.69
Proposed sensor	4.87	5.85	6.80	4.57

**Table 3:** The first six natural frequencies.

Mode	1	2	3	4	5	6
Frequency (Hz)	79.06	79.17	127.9	407.9	424.5	428.2

**Table 4:** Variable constraints.

	*m* (mm)	*b* (mm)	*r* (mm)
Maximum	280	220	120
Minimum	200	180	80

**Table 5:** The last design variables and corresponding objective function.

*r* (mm)	*b* (mm)	*m* (mm)	Workspace	GSDI
80.000	187.448	272.140	5342	1.753
80.001	187.280	271.858	5305	1.752
80.350	187.112	272.871	5428	1.756
80.339	187.093	272.790	5413	1.755
80.001	187.282	271.856	5304	1.752
80.000	187.265	271.922	5317	1.752
85.916	180.461	279.073	6242	1.796
80.436	186.805	273.378	5523	1.756
80.353	187.090	272.798	5415	1.755
85.955	180.000	279.688	6350	1.797
80.007	187.101	271.914	5326	1.752
80.009	187.259	271.936	5319	1.752
80.000	187.453	272.144	5343	1.753
80.000	187.435	272.170	5344	1.754
80.009	187.272	271.907	5314	1.752
80.000	186.675	271.801	5340	1.752
80.000	186.680	271.793	5338	1.752
86.003	180.154	279.581	6327	1.797
80.000	187.280	271.860	5305	1.752
80.350	187.111	272.870	5428	1.756
